# Chick sexing based on the blood analysis using Raman spectroscopy

**DOI:** 10.1038/s41598-024-65998-y

**Published:** 2024-07-11

**Authors:** Sana Matsumoto, Akane Ogino, Kai Onoe, Juichiro Ukon, Mika Ishigaki

**Affiliations:** 1 Institute of Agricultural and Life Sciences, Academic Assembly, Shimane University, 1060 Nishikawatsu, Matsue, Shimane 690-8504 Japan; 2 NABEL Co., Ltd., 86 Morimoto-Cho, Nishikujo, Minami-Ku, Kyoto, 601-8444 Japan; 3 UKON Craft Science Ltd., 106-4, Fukakusa-Shhinmon-Jotyo, Fushimi-Ku, Kyoto, 612-8436 Japan

**Keywords:** Analytical chemistry, Raman spectroscopy

## Abstract

Efforts are underway to develop technology for automatically determining the sex of chick embryos, aimed at establishing a stable and efficient poultry farming system while also addressing animal welfare concerns. This study investigated the possibility of chick sexing through blood analysis using Raman spectroscopy. Raman spectra were obtained from whole blood and its constituents, such as red blood cells (RBCs) and blood plasma, collected from chicks aged 1–2 days, using a 785-nm excitation wavelength. Principal component analysis (PCA) revealed statistically significant sex-dependent spectral variations in whole blood and RBCs, whereas blood plasma showed less clear dependency. These spectral differences between male and female chicks were attributed to differences in the proportion of spectral components from oxygenated (oxy-) and deoxygenated (deoxy-) RBCs, with males exhibiting a slightly stronger contribution of oxy-RBCs compared to females. This reflects the higher oxygen affinity of hemoglobin (Hb) in males compared to females. A model for discriminating chick sex was built using the ratios of certain Raman band characteristics of oxy-RBCs and deoxy-RBCs, achieving a sensitivity of 100%. This spectroscopic method holds promise for developing technology to discriminate the sex of early chicken embryos in ovo by detecting differences in oxygen saturation of RBCs based on sex.

## Introduction

In 2019, Japan maintained a high self-sufficiency rate for eggs at 96%^1^. The hens responsible for egg production are called laying hens. Notably, male chickens do not contribute to egg laying, rendering them economically nonviable. Consequently, approximately seven billion male chicks are annually killed worldwide shortly after hatching, presenting both economic losses and significant animal welfare concerns. Compounding this issue is the challenge of determining the gender of chicks, as they lack external genitalia, making their visual identification difficult^2^. Currently, their gender determination relies on manual inspection, assessing their wing shape and the presence of anal protrusions, which require skilled labor and prone to inefficiencies. In an era marked by rapid advancements in artificial intelligence and automation across industries, it is imperative for the poultry industry to automate the sexing process of early chicken embryos in ovo. Development of such technology is crucial, aligning with the trend toward increased automation and autonomy in modern industries.

Raman spectroscopy is a powerful tool expected to revolutionize chick sex determination because it can provide information about biological molecules, such as proteins, lipids, saccharides, and DNA/RNA, in a nondestructive and noninvasive manner^3,4^. Thus, Raman spectroscopy is suitable for analyzing living organisms, leading to its widespread adoption across various biological and medical applications^5–12^. Especially, hemes as chromophores exhibit distinctive electric absorption bands within the ultraviolet–visible (UV–vis) wavelength region^13–18^, thereby eliciting a strong resonance enhancement of Raman scattering selectively for hemes^19,20^. Thus, resonance Raman and preresonance Raman spectroscopies have found application in blood analysis^19,20^, with some studies exploring its utility in chick sexing^21,22^. Galli et al. reported the in ovo sexing of chicken eggs via Raman spectroscopy, analyzing embryo blood spectra from vitelline blood vessels under a 785-nm excitation wavelength. Leveraging linear discriminant analysis (LDA) and genetic algorithm, they achieved a 90% classification accuracy in the chick sexing process^21^. They also explored sexing techniques for turkey poults and chicken eggs using Fourier transform infrared spectroscopy and fluorescence spectroscopy, respectively^23,24^. Harz et al. determined bird gender using UV-resonance Raman spectroscopy with a 244-nm excitation through feather pulp analysis. By assigning differences in resonance Raman spectra corresponding to DNA bases and employing supervised classification via support vector machine (SVM), they achieved 95% accuracy in gender determination^22^. In a different context focusing on humans, Sikirzhytskaya et al. utilized Raman spectroscopy with a 785-nm excitation wavelength to determine the gender of adults from dry bloodstains^25^. Despite similar Raman spectra patterns across genders, leveraging statistical methods such as SVM, artificial neuron network, and genetic algorithm facilitated gender discrimination with ~ 98% accuracy^25^.

Herein, the possibility of determining the sex of chicks through blood analysis was investigated using Raman spectroscopy, aiming to establish a method for chicken embryo sexing. Raman spectra from whole blood and its components—red blood cells (RBCs) and blood plasma—obtained from 1–2-day-old chicks underwent PCA and partial least square (PLS) regression analysis. To explore the causes for spectral differences depending on chick sex, resonance and preresonance Raman spectra of oxygenated (oxy-) and deoxygenated (deoxy-) RBCs were also investigated in detail. Models for discriminating chick sex were then constructed using LDA and original multiple regression, calculating ratios of characteristic Raman bands with notable differences between male and female chicks (*p*-values of the Student’s *t*-test were smaller than 0.05), particularly those related to the oxy-RBC and deoxy-RBC bands. The findings of this study showed the feasibility of chick sex discrimination using Raman spectroscopy combined with statistical approaches. Further endeavors will focus on developing this method for early chicken embryo sexing in ovo to solve the serious social concern for animal welfare.

## Materials and methods

### Chicken eggs

Fertilized eggs of *Julia light* breed chickens were purchased from Japan Layer Co. Ltd (Gifu, Japan). After a 2-day storage period in a cryocooler at 18 °C, the eggs were transferred to an incubator (Baby B type, Showa Furanki Co. Ltd., Saitama, Japan) maintained at 39–40 °C with automated rotation occurring once per hour. The day on which the eggs were put in the incubator was defined as D0, with hatching occurring on D20–D21. Chick sex was determined through dissection to examine intraperitoneal reproductive organs after blood collection or by analyzing feather morphology.

All the experiments adhered to the fundamental guidelines for ethical conduct in animal research, as outlined by Animal Research: Reporting of In Vivo Experiments (ARRIVE) guidelines and the Ministry of Education, Culture, Sports, Science and Technology in Japan. The present study was approved by the Ethics Committee of Shimane University in Japan (MA3-12).

### Blood samples

Blood samples were collected from chicks on the first or second day after hatch through cardiac puncture using blood collection tubes containing heparin sodium. The blood collection was carried out under the loss of consciousness by CO_2_ gas within a sealing container, and chicks were sacrificed by keeping them in the airtight container filled with CO_2_ gas. The obtained blood samples (male: 8 and female: 8) were measured intact or after separation into blood cells and blood plasma via centrifugation (6000 rpm for 30 s). Blood plasma was separated as the supernatant, while the remaining samples underwent multiple washes with physiological saline (0.01 mol/L Phosphate Buffered Saline, 164–18,541, Wako Pure Chemical Industries, Ltd, Japan) to obtain blood cells. To obtain reference Raman spectra for oxy- and deoxy-RBCs, the airflow of oxygen and nitrogen was directed over the samples using a straw for one min each, respectively, that were newly collected from seven male and eight female chicks. The collected blood samples were immediately stored in a refrigerator, and Raman measurements were performed at room temperature soon after taking them out from the refrigerator within 24 h after blood collection. Raman spectra of oxy- and deoxy-RBCs were also obtained for reference purposes. The reference spectral data are depicted in Supplementary Information (SI) 1.

### Raman measurements

Raman measurements of the blood samples were conducted using the XploRA INV microscope Raman system (HORIBA Ltd., Kyoto, Japan), featuring a 785-nm diode laser, a spectrometer, a 600- or 1200-gr/mm grating, a charge-coupled device camera, and either a 60 × water emersion objective lens (Plan Apochromat, NA = 1.20, Nikon) or a 10 × objectiveens (TU Plan Fluor, NA = 0.3, Nikon) mounted on a microscope (Eclipse Ti2 Inverted Microscope, Nikon Co., Tokyo, Japan). Blood samples were placed on quartz dishes, with Raman spectra acquired at 1–2 points per sample, changing the measurement locations. To conduct the analysis regardless of the time elapsed after blood collection, Raman measurements were performed in the order of sampling, that was in a manner regardless of the chick sex. The measured Raman spectra were calibrated using the silicon peak at 520 cm^−1^. Raman spectra were recorded within the 1800–600 cm^−1^ region with exposure times of either 30 s and 10 scans or 15 s and 4 scans. The laser power at the sample point was about 40 mW. The obtained Raman spectra were preprocessed via background subtraction, baseline correction with fifth-order polynomial fitting, and normalization using the area of spectra within the 1800–600 cm^−1^ region. The selection of the excitation wavelength appropriate for chick sexing through blood analysis was first conducted. Detailed procedures and discussions are explained in SI1 (Figures S1, S2, and S3, and Table S1).

For spectral analysis, the chemometrics software Unscrambler X 10.3 (Camo Analytics, Oslo, Norway) and OriginPro 2022 (OriginLab Corporation, Massachusetts, USA) were used. PCA method extracted a set of orthogonal principal components (PCs) to account for the variances within the spectral dataset. The results of PCA are typically discussed in terms of scores and loadings of PCs^26^.

LDA was conducted using the score vectors derived from PCA (PCA–LDA)^27^, employing the leave-one-out cross-validation method. In this method, one Raman spectrum was withheld for validation, while the LDA model for sexing was built using the remaining spectral data. Then, the generated model was used to predict the sex of the validated data. This process was repeated until all spectra were discriminated.

PLS regression analysis was applied to construct a linear regression model between measurement data ($X$*)* and a response variable ($Y$), such as the concentration and density of a material^11,28^. If the measured data include the components represented by the response variable, regression coefficients ($B$) exist that satisfy Eq. (1).

1 $$Y = XB + E$$

Here, $E$ is a residual, and all variables are generally expressed as matrices. $X$ and $Y$ are factorized into optimal loadings and scores to satisfy Eq. (1). Thus, the loadings and scores express how the data are projected onto the model components. For a linear regression model with high calibration accuracy, the squared correlation coefficient (*R*^*2*^) is close to 1. In this study, the concentration profile *Y* was not known. Thus, PLS analysis was conducted by giving artificial concentration profiles to extract the components that were correlated with the concentration profiles, and a calibration model was built using the leave-one-out cross (LOOC) validation method^29^. The LOOC method consisted of excluding one spectrum for use in validation, and the calibration model was built using the remaining data. The excluded spectrum was applied to the calibration model, and the predicted residual was calculated. This process was repeated until all the spectra had been excluded once. The accuracy of the models was assessed in terms of the *R*^*2*^ value.

### Statistical analysis

The Student’s *t*-test was employed to compare datasets from two groups to identify significant differences between males and females. A threshold *p*-value of 0.05 was utilized to determine the significance of observed differences.

## Results and discussion

### Raman spectra of blood obtained from 1–2-day-old chicks

**Figure 1:**
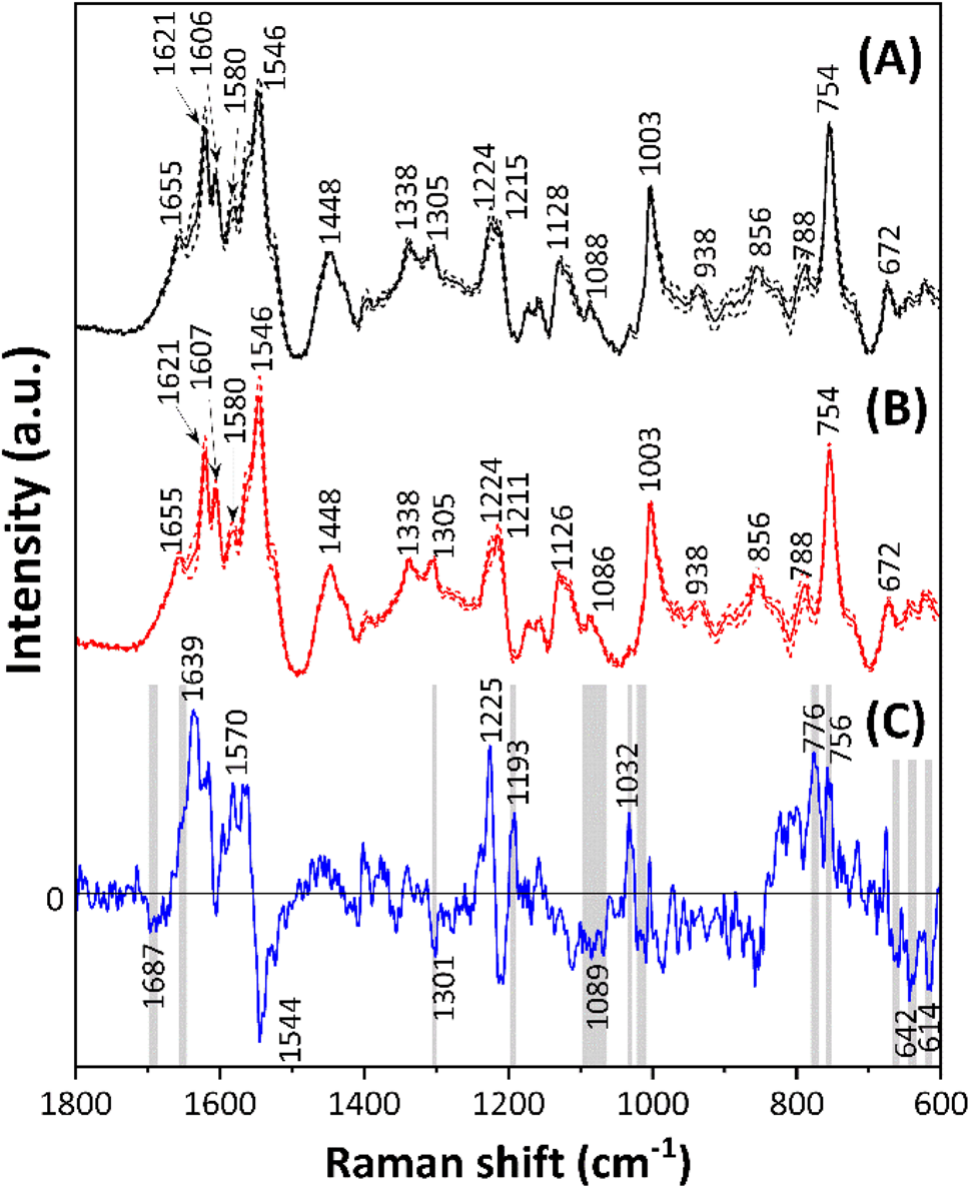
Mean Raman spectra and one standard deviation (1SD) in the 1800–600 cm^−1^ region for blood from (**A**) male (*n* = 14) and (**B**) female (*n* = 16) chicks aged 1–2 days. The solid and dotted lines express the mean spectra and mean spectra ± 1SD, respectively. (**C**) Difference spectra calculated as the mean spectra of males minus that of females.

Figure 1A and B show the mean Raman spectra, along with one standard deviation, within the 1800–600 cm^−1^ region for blood obtained from 1–2-day-old male (*n* = 14) and female (*n* = 16) chicks. These blood samples comprise RBCs, white blood cells, platelets, and blood plasma. The spectra mainly exhibit mixed properties arising from bands attributed to porphyrin, a constituent of hemes in RBCs, and lipoproteins. The detailed explanations for the spectral properties of blood samples are discussed in SI1. General proteinaceous bands are evident at 1665 and 1003 cm^−1^, corresponding to amide III and the ring breathing mode of phenylalanine, respectively. In addition, bands associated with C–C, C=C, and C–H components in lipoproteins and porphyrin are observed at 1621, 1606, 1448, 1088, 938, and 856 cm^−1^. Characteristic bands originating from porphyrin are detected at 1580, 1546, 1338, 1305, 1224, 1215, 1128, 788, 754, and 672 cm^−1^. The detailed band assignments are summarized in Table 1^30–34^.

**Table 1:** Band assignments and local coordinates for whole blood from young chickens aged 1 to 2 days recorded using 785-nm excitation^30–34^.

Band position (cm^-1^)	Assignment	Local coordinate
1655	Amide I	
1621	ν(C = C)_vinyl_	ν(C_a_ = C_b_)
1606	ν(C = C)_vinyl_	ν(C_a_ = C_b_)
1580	ν_37_, phenylalanine	ν(C_α_C_m_)_asym_
1546	ν_11_	ν(C_β_C_β_)
1448	δ(CH_2_/CH_3_)	
1338	ν_41_	ν(pyr half-ring)_sym_
1305	ν_21_	δ(C_m_H)
1224	ν_13_ or ν_42_	δ(C_m_H)
1215	ν_5_ + ν_18_	δ(C_m_H)
1128	ν_5_	ν(C_β_–methyl)
1088	δ(CH_2_/CH_3_)	
1003	phenylalanine	
938	ν(C–C)	
856	ν(C–C)	
788	ν_6_	ν(pyr breathing)
754	ν_15_	ν(pyr breathing)
672	ν_7_	δ(pyr deform) _sym_

ν: stretching mode, δ: bending mode.

The difference spectrum, calculated as the mean spectrum of females subtracted from that of males shown in Fig. 1A and B, showed peaks at 1639, 1570, 1225, 1193, 1032, 776, and 756 cm^−1^ in the positive domain and at 1687, 1544, 1301, 1089, 642 and 614 cm^−1^ in the negative domain (Fig. 1C). Gray-shaded areas denote wavenumber regions with significant differences in spectral intensities between males and females, as determined through the *t*-test (*p* < 0.05). Notable observations include a band at 1657 cm^−1^ in the 1700–1550 cm^−1^ region, peaks at 1301, 1193, 1089, and 1032 cm^−1^, and peaks in the 760–750 cm^−1^ region characteristic of the porphyrin (Fig. 1C).

**Figure 2:**
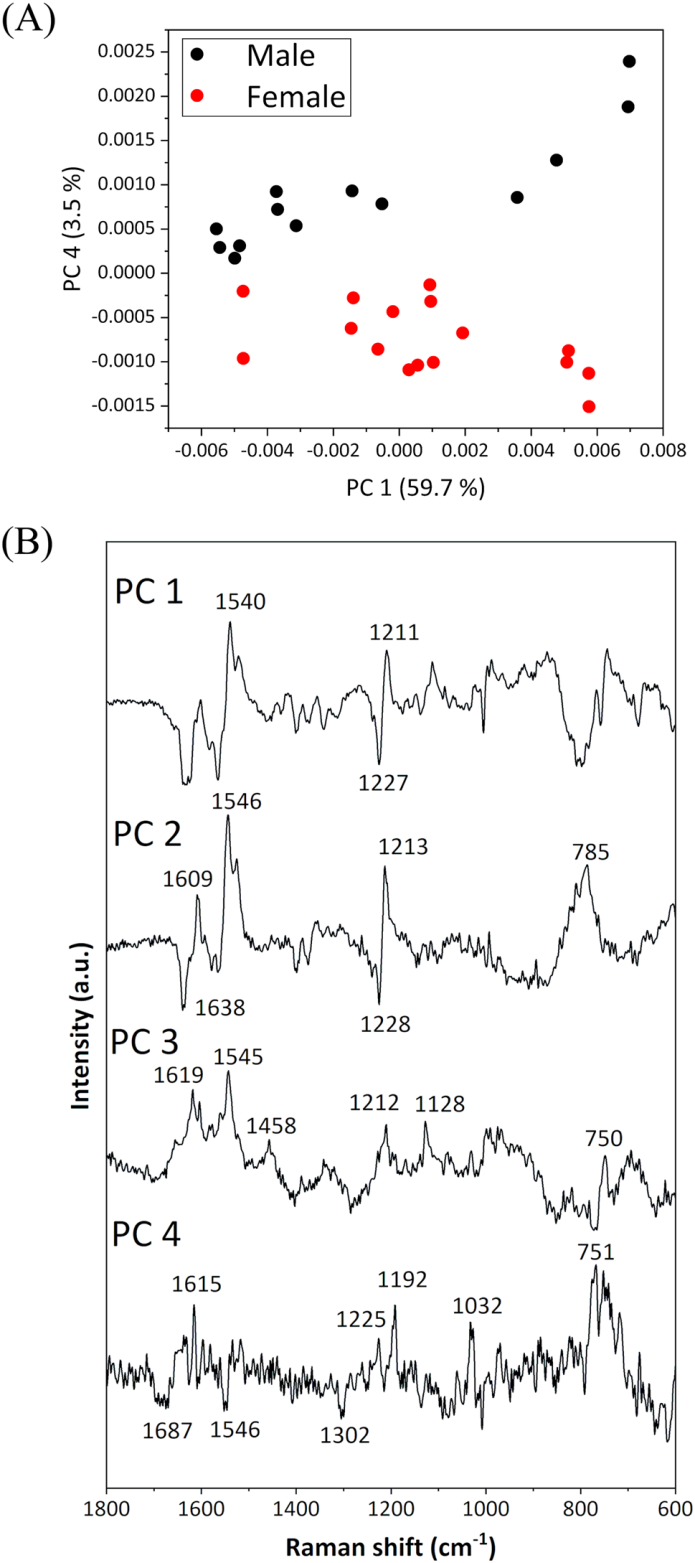
(**A**) Score plots of PCA (PC 1 vs. PC 4) derived from the Raman spectral dataset of blood from male and female chicks. (**B**) Loading plots of PC 1, PC 2, PC 3, and PC 4.

PCA analysis was conducted for these spectral data, including males and females. Figure 2A shows score plots of the PC 1 vs. PC 4, revealing a distinct classification of male and female datasets into two groups based on PC 4. In the loading plots of PC 1, PC 2, and PC 3, peaks corresponding to pyrroles were extracted. Conversely, in the loading plot of PC 4, peaks were extracted at 1687, 1615, 1546, 1302, 1225, 1192, 1032, and 751 cm^−1^, corresponding to wavenumber regions with significantly different spectral intensities between males and females, as defined using the *t*-test (Fig. 1C). Thus, distinguishing between male and female 1–2-day-old chicks based on the Raman spectra of blood is deemed feasible.

### Raman spectra of blood components: blood cells and blood plasma obtained from 1–2-day-old chicks

**Figure 3:**
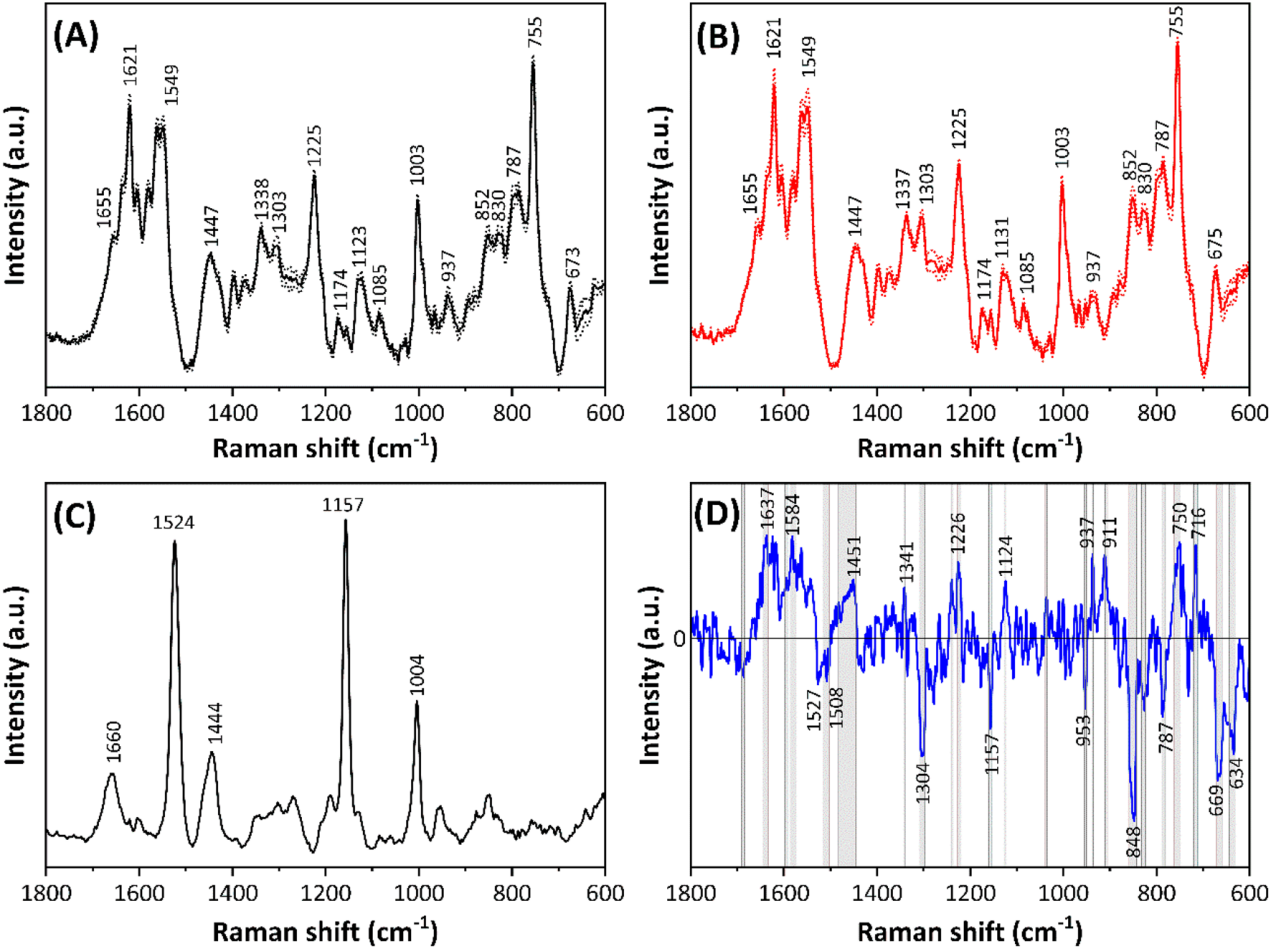
Mean Raman spectra of RBCs from (**A**) male (*n* = 12) and (**B**) female (*n* = 11) chicks, and (**C**) blood plasma obtained from 1–2-day-old chickens. The solid and dotted lines express the mean spectra and mean spectra ± 1SD, respectively. (**D**) Difference spectra of RBCs calculated as the mean spectra of males minus that of females.

To delve into the specific blood component responsible for sex-dependent properties, separate Raman spectra of blood cells and plasma obtained through centrifugation were analyzed. Figure 3A and B depict the mean Raman spectra of blood cells for males (*n* = 12) and females (*n* = 11), respectively. Notably, both spectra show intense bands at 1621, 1549, 1225, 755, and 673 cm^−1^ attributed to the porphyrin, reflecting the spectral properties of RBCs. Wood et al. reported the Raman spectral differences between oxy- and deoxy-RBCs in human blood using a 785-nm excitation^30^. They concluded that the most intense band in deoxy-RBC spectra is observed at 1549 cm^−1^ rather than 1621 cm^−1^. The spectral patterns of blood cells from males and females shown in Fig. 3A and B reveal the characteristic pattern corresponding to oxy-RBCs.

Figure 3C shows the mean Raman spectra of blood plasma (*n* = 23), including both males and females, derived from the same blood samples used for blood cells in Fig. 3A and B. Intense bands observed at 1524 and 1157 cm^−1^ correspond to C=C and C–C stretching vibrational modes of carotenoids^35–37^, in addition to bands located at 1660, 1444, and 1004 cm^−1^ attributed to proteins (Table 1). The yellowish hue of blood plasma is attributed to carotenoids, such as lycopene and β-carotene, obtained from their dietary sources^38^. Figure 3D depicts the difference spectrum of blood cells, calculated as the mean spectrum of females subtracted from that of males. Wavenumber regions with significant differences in spectral intensities between males and females, as determined using the *t*-test (*p* < 0.05), are highlighted in gray. Numerous peaks are observed in both positive and negative directions, with a detailed discussion provided in subsequent sections.

**Figure 4:**
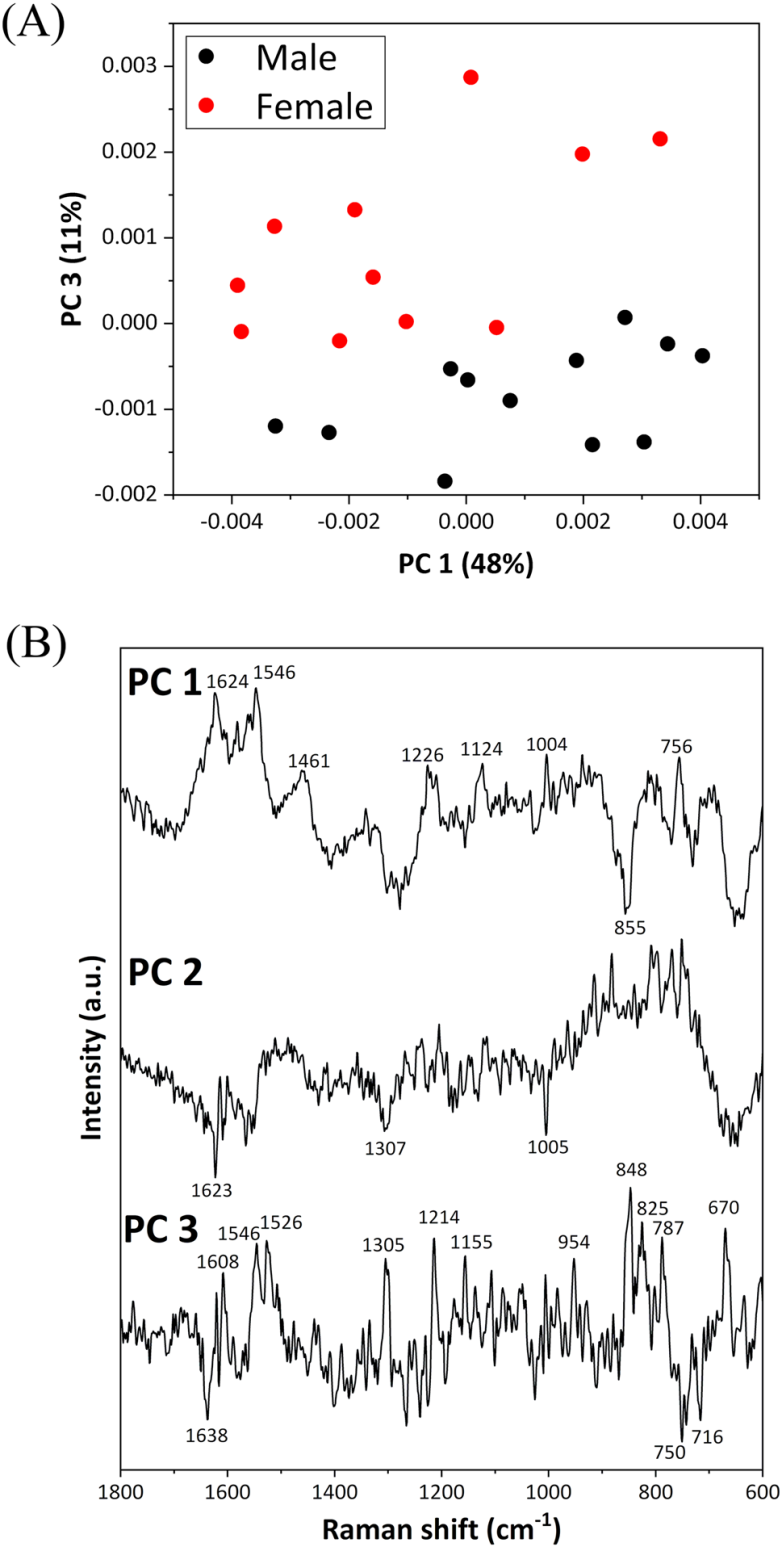
(**A**) Score plots of PCA (PC 1 vs. PC 3) calculated for the dataset of blood cells, including males and females. (**B**) Loading plots of PC 1, PC 2, and PC 3.

PCA was employed to analyze the Raman spectral dataset of blood cells to study the differences depending on chick sex. The PCA score plots (PC 1 vs. PC 3) revealed PC 3 as the discriminating factor between males and females (Fig. 4A). In the loading plot of PC 3, peaks were observed at 1546, 1526, 1305, 1214, 848, and 670 cm^−1^ in the positive domain and at 1638, 750, and 716 cm^−1^ in the negative domain (Fig. 4B). Notably, the difference spectrum of blood cells (Fig. 3D) exhibited a similar pattern to their loading spectrum (Fig. 4B), albeit with reversed positive and negative values. Thus, the loading plot effectively captured the spectral differences of RBCs depending on chick sex.

Similarly, PCA was applied to the blood plasma dataset, including Raman spectra of males (*n* = 16) and females (*n* = 17). However, score plots for all PCs did not show separation based on sex-related spectral variations. Thus, the spectral disparities in blood depending on chick sex were primarily attributed to differences in RBCs.

### Raman spectra of oxy- and deoxy-RBCs obtained from 1–2-day-old chicks

**Figure 5:**
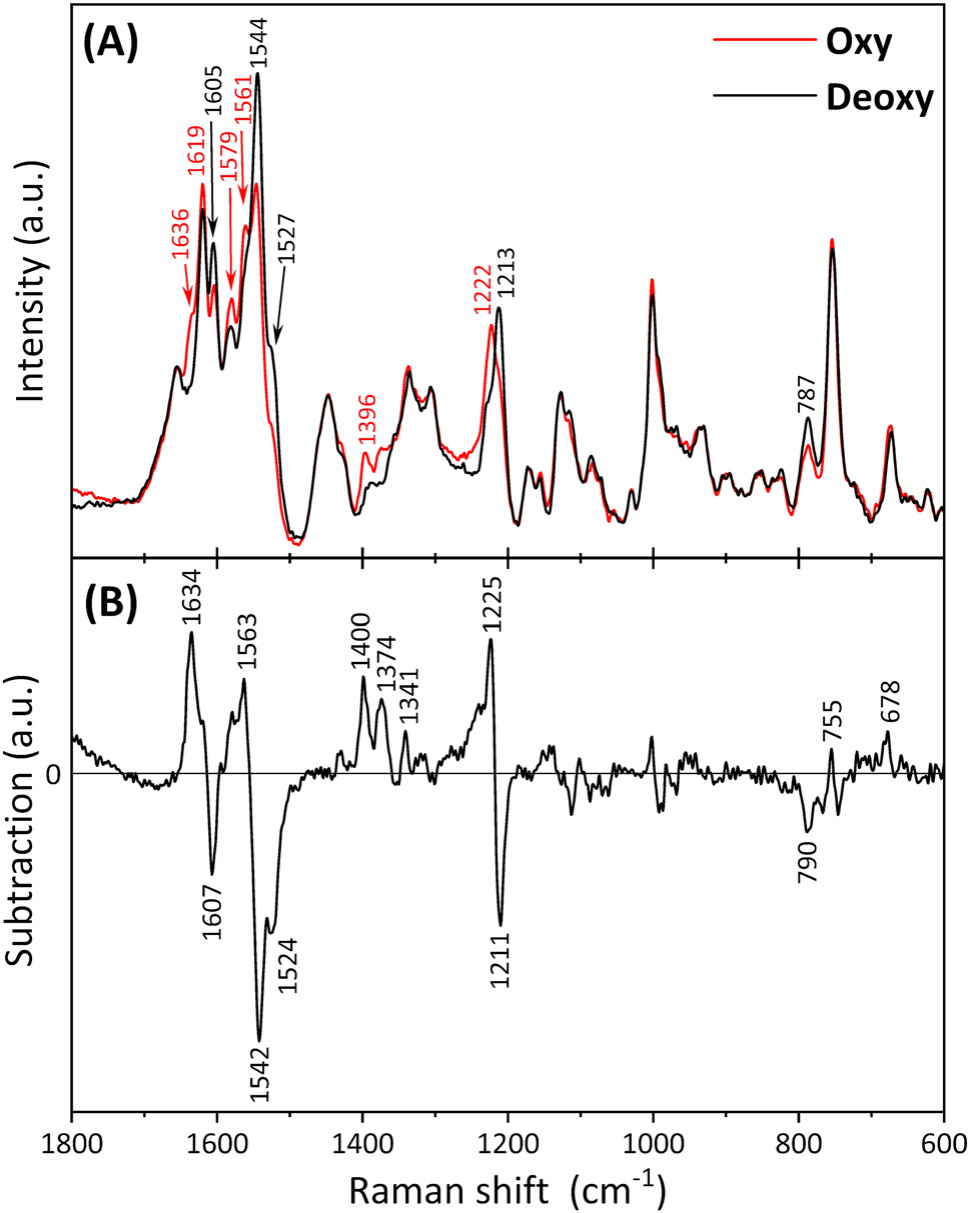
(**A**) Mean Raman spectra of oxy- and deoxy-RBCs from 1–2-day-old chicks. (**B**) Subtraction spectra of (**A**) calculated as the spectrum of oxy-RBCs minus that of deoxy-RBCs.

To further investigate the origins of the differences in Raman spectra for RBCs between males and females, Raman spectra for oxy- and deoxy-RBCs of 1–2-day-old were obtained. Figure 5A shows Raman spectra within the 1800–600 cm^−1^ region for oxy- and deoxy-RBCs. A comparison of these two spectra revealed distinct differences in certain bands within the 1650–1500 cm^−1^ region depending on the states of oxy- or deoxy-RBCs. In the deoxy-RBC spectrum, as previously noted, the band intensity at 1544 cm^−1^ appeared stronger than that at 1619 cm^−1^. Conversely, in the oxy-RBC spectrum, the intensities of these two bands were almost comparable. Furthermore, the band intensities at 1636, 1579, and 1561 cm^−1^ were stronger in oxy-RBCs than in deoxy-RBCs, while a shoulder band observed at 1527 cm^−1^ was more intense in deoxy-RBCs. Subtraction spectrum, calculated as the spectrum of oxy-RBCs minus that of deoxy-RBCs, revealed that the more intense bands appeared in the spectra of oxy- and deoxy-RBCs in positive and negative domains, respectively (Fig. 5B). In the lower wavenumber region, the band at 1396 cm^−1^ was exclusively observed in the spectrum of oxy-RBCs, while the band at 787 cm^−1^ was more intense in the spectrum of deoxy-RBCs. Moreover, the relative band intensities at 1222 and 1213 cm^−1^, appearing as shoulder bands for each other, seemed to differ depending on the state of oxy- or deoxy-RBCs. The peaks at 1637, 1584, 1341, 1226, and 750 cm^−1^ in the positive domain of the loading plot of PC 3 (Fig. 3D) aligned well with the characteristic bands for oxy-RBCs shown in Fig. 5B, while the negative peaks at 1527 and 787 cm^−1^ in the loading plot of PC3 (Fig. 3D) corresponded to the bands for deoxy-RBCs shown in Fig. 5B. These results suggested that the PC 3 reflects the spectral differences attributed to the redox states of RBCs, with males having a higher proportion of oxy-RBCs.

### Quantitative analysis of Raman spectra to extract blood components with different concentrations depending on chick sex

**Figure 6:**
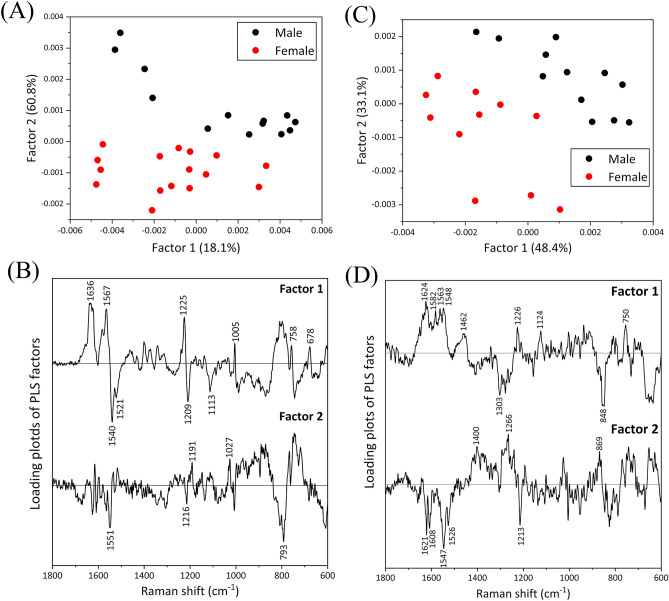
Score plots of PLS for Factors 1 vs. 2 calculated for the Raman spectral dataset of (**A**) whole blood and (**C**) blood cells. Loading plots of Factors 1 and 2, extracted as the spectral components with the concentration gradient given for (**B**) whole blood and (**D**) blood cells.

To perform a quantitative analysis of the concentration differences based on the chick sex, PLS regression analysis was conducted. The concentration profile about arbitrary blood components defined as (male, female) = (2, 1) was given as an input parameter to conduct PLS regression analysis, and a calibration model reproducing the given concentration profile was built using the LOOC method^29^. Figure 6A and B depict the score and loading plots of PLS (Factors 1 and 2, respectively) generated for the dataset of whole blood. Factors 1 and 2 were identified as the main spectral components reflecting concentration profile. The *R*^*2*^ showed high accuracies for calibration and validation, with values of 1.0 and 0.84, respectively, by including up to six factors. In the loading plot of Factor 1, distinct bands were observed at 1636, 1567, and 1225 cm^−1^ in the positive domain and at 1540, 1521, and 1209 cm^−1^ in the negative domain, representing characteristic bands of oxy- and deoxy-RBCs, respectively, as shown in Fig. 5A. Conversely, in the loading plot of Factor 2, bands characteristic of deoxy-RBCs appeared at 1551 and 793 cm^−1^ in the negative domain. These results indicated that the spectral components derived from oxy- and deoxy-RBCs in blood were stronger and weaker, respectively, in males compared to females.

Similarly, PLS analysis was performed on the dataset of Raman spectra for RBCs. Factors 1 and 2 of PLS were extracted as the spectral components corresponding to the given concentration gradient. The *R*^2^ value for validation and calibration reached 0.93 and 0.73, respectively, with up to three factors. While the accuracy in building the quantitative model for blood component concentrations appeared slightly reduced, it can be affirmed that the quantitative accuracy for biological samples was sufficiently maintained. The score and loading plots of Factors 1 and 2 are shown in Fig. 6C and D, respectively. In the loading plot of Factor 1, the spectral component for males exhibited stronger contributions to the positive bands at 1624, 1582, 1563, and 750 cm^−1^ than those for females. Focusing on the wavenumber region above 1500 cm^−1^, the band intensity at 1624 cm^−1^ was stronger than that at 1548 cm^−1^, indicating that Factor 1 likely reflected the Raman signal derived from oxy-RBCs. Conversely, in the loading plot of Factor 2, an opposite trend was observed in the wavenumber region above 1500 cm^−1^ compared to Factor 1; the band intensity at 1547 cm^−1^ was more intense than that at 1621 cm^−1^. These results suggest that Factor 2 expressed the contribution of deoxy-RBCs.

In this way, a model for quantitative evaluation was constructed, assuming that one component was present in higher concentration in males than in females, to extract the spectral components contributing more precisely to chick sex discrimination in the samples of whole blood and RBCs. As results, it was reconfirmed that chick sexing using the Raman spectra of whole blood and RBCs was accomplished based on the characteristic Raman bands attributed to oxy- and/or deoxy-RBCs.

## Discussions

The preceding sections indicated that differences in Raman spectra based on the chick sex were reflective of the states of oxy- and deoxy-RBCs. To study the origin of the different states of RBCs in detail, RBCs from males and females were independently measured after conversion into oxy- and deoxy-states for RBCs using 532- and 785-nm excitations in SI 1. Figure S3A shows the Raman spectra of oxy- and deoxy-RBCs obtained via a 532-nm excitation, while Fig. S3B depicts those obtained via a 785-nm excitation, showcasing similar spectral patterns shown in Fig. 5A. Notably, no discernible spectral differences based on chick sex were evident upon initial observation for both 532- and 785-nm excitations.

**Figure 7:**
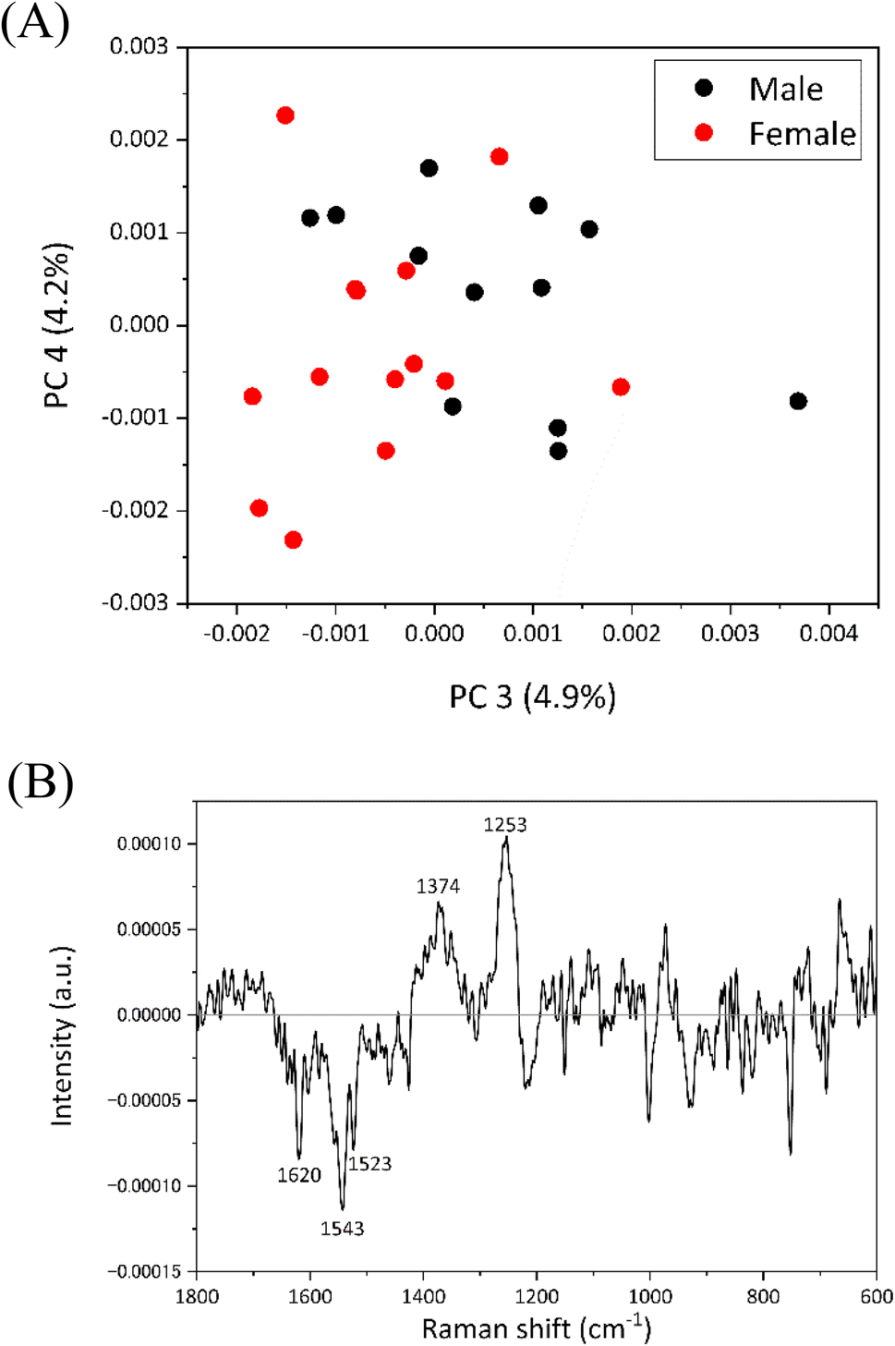
(**A**) Score plots of PCA (PC 3 vs. PC 4) for the dataset of oxy-RBCs measured using 785-nm excitation. (**B**) A difference spectrum of the mean spectrum of oxy-RBCs for males from the mean spectrum calculated from the spectral dataset of oxy-RBCs, including males and females measured using a 785-nm excitation.

PCA was conducted independently for the excitation wavelength and the states of oxy- and deoxy-RBCs in the Raman spectral dataset, including male and female samples. The score plots of PCA for deoxy-RBCs measured using a 785-nm excitation wavelength, did not exhibit any split patterns indicating differentiation based on chick sex. Conversely, for oxy-RBCs, the data tended to be roughly classified into two groups solely based on sex when using a 785-nm excitation, albeit with some overlapping (Fig. 7A). To extract the different spectral components depending on sex, the mean Raman spectrum of oxy-RBCs was subtracted from the mean spectrum of oxy-RBCs for males or females. Figure 7B depicts the subtraction spectrum for males, while the subtraction spectrum for females showed opposite patterns to those for males along the *y*-axis. Notably, similar spectral pattern was observed in the higher wavenumber region (> 1200 cm^−1^) of Fig. 7B to those of Factor 2 of the PLS analysis (Fig. 6B). This spectrum was extracted as the spectral component characteristic for deoxy-RBCs and showed lower contribution in males compared to females for the whole blood samples. Therefore, the result consistently showed a higher contribution of oxy-RBCs in males compared to females.

In the case of a 532-nm excitation, the data did not exhibit based on chick sex. The different outcomes depending on the excitation wavelength may stem from the standardization of spectral intensity. With a 532-nm excitation, both oxy- and deoxy-RBC spectra were equally enhanced by the resonance effect^14–18^, resulting in prominent variations in their spectral patterns. Because the spectra were normalized based on each spectral area, the spectral variations caused by the states of oxy- and deoxy-RBCs influenced spectral intensities on the whole, thereby hindering the extraction of sex-related differences in RBCs. These results collectively suggest that the molecular structure of RBCs does not differ between males and females, but rather, their degrees of oxygen saturation vary. Moreover, the excitation wavelength appropriate for chick sexing was found to be 785 nm, with sex being identifiable based on the degree of oxygen saturation.

Several previous studies support the results of this study. For instance, the oxygen affinity of Hb for healthy young nonsmokers is lower in females than in males, as revealed by the higher partial pressures of oxygen for females when 50% of Hb is saturated^39^. Eaton et al. reported a faster rate of oxygen release from women’s blood compared to men’s blood^40^. Regional cerebral oxygen saturation measured using near-infrared spectroscopy was considerably lower in females than in males^41^. Thus, the basis for distinguishing chick sex lies in detecting the degree of oxygen saturation, with male blood likely possessing a higher oxygen affinity of Hb than female blood.

### Hematological test

To investigate potential differences in blood component concentrations related to RBCs based on chick sex, heparinized blood samples (male: 27, female: 26) were outsourced to Hoken Kagaku, Inc., Kanagawa, Japan, for hematological testing. The tests examined six parameters for red blood cells: RBC count, Hb content (Hgb), hematocrit value (Hct), mean corpuscular volume, mean corpuscular Hb, and mean corpuscular Hb concentration. The averages and standard errors for each hematological test are summarized in Table 2. There were no significant gender differences observed for any blood component. These results confirm that variations in chick blood components based on sex are attributed to differences in the bonding of Hb with oxygen atoms rather than variations in Hb concentrations.

**Table 2:** Hematological test results for six items.

	RBC (× 10^4^/μL)	Hgb (g/dL)	Hct (%)	MCV (fL)	MCH (pg)	MCHC (%)
Male	230.4 ± 3.2	7.0 ± 0.1	30.3 ± 0.4	131.0 ± 0.4	30.4 ± 0.4	23.1 ± 0.3
Female	232.7 ± 3.1	7.2 ± 0.2	30.3 ± 0.4	129.7 ± 0.7	30.9 ± 0.5	23.7 ± 0.3
*p*-value	0.61	0.31	0.97	0.11	0.40	0.17

### Model building for discriminating chick sex

In the preceding section, it was demonstrated that the sex of chicks could be discriminated through the characteristic Raman bands associated with oxy- and deoxy-RBCs. Thus, models for chick sexing were constructed, and their accuracies were verified.

First, PCA–LDA was conducted on the dataset comprising whole blood and RBCs, using the LOOC technique. During validation, one sample was held out as a validation sample, and the LDA model was built using the remaining samples. Subsequently, this model was used to predict the class of the validated sample, and this process was repeated until all spectra were classified. The classification encompassed 30 samples (male: 14, female: 16) for whole blood and 23 samples (male: 12, female: 11) for RBCs. The model was built based on PC-4 scores for whole blood and PC-3 scores for RBCs, which were selected as the components with spectral information for chick sexing, as shown in Figs. 2B and 4B, respectively. Table 3A and B provide a list of the true sex and the predicted sex of chicks validated using PCA–LDA for whole blood and RBCs, respectively. In both cases, one female sample was misclassified as male. The prediction accuracies for whole blood resulted in 96.7%, and for RBCs, it was 95.7%. Thus, PCA–LDA demonstrated the capability to distinguish between males and females with high accuracy for both whole blood and RBCs.

**Table 3:** List of true sex of chicks and their prediction sex validated using PCA–LDA for (A) whole blood and (B) red blood cells.

Whole blood		Predicted sex		
		Male	Female	Total
(A)				
True sex	Male	14	0	14
	Female	1	15	16
Total		15	15	96.7%

Red blood cells		Predicted sex		
		Male	Female	Total
(B)				
True sex	Male	12	0	12
	Female	1	10	11
Total		13	10	95.7%

Second, it was verified whether it is feasible to easily distinguish between males and females based on the intensity of narrowed bands with significant differences between the sexes (*P* < 0.05). The focus during model building was on the observation that the Raman spectra of RBCs for males and females showed similar spectral patterns; there were no bands unique to either males or females, and numerous common bands were observed in the spectra of both oxy- and deoxy-RBCs. Consequently, discerning the sex of chicks solely by detecting a specific Raman band was deemed impractical.

Thus, attention was directed toward the bands at 787 and 1640 cm^−1^, which appeared in positive and negative directions, respectively, in the difference spectra of RBCs between males and females (Fig. 4B). The band intensity at 787 cm^−1^ was stronger in the Raman spectra of deoxy-RBCs, while at 1640 cm^−1^, it was stronger in the spectra of oxy-RBCs (Fig. 5A). These bands were identified to have significant differences between males and females based on *t*-test results. The box plots of the band intensities are shown in Figs. S4A and S4B. The band intensities at 787 and 1640 cm^−1^ were stronger for females and males, respectively, which were strongly detected in the spectra of deoxy- and oxy-RBCs (Fig. 5A). The *p*-values for these bands were 0.016 (787 cm^−1^) and 3.8 × 10^−3^ (1640 cm^−1^), indicating significant differences between the sexes. Consequently, the intensity ratio of these bands (*I*_787_/*I*_1640_) was calculated to define a parameter that emphasizes the differences depending on the sex. Figure S4C exhibits the box plot of this ratio, resulting in an improved *p*-value of 1.1 × 10^−3^ by considering the intensity ratio of the bands compared to individual band intensities.

**Figure 8:**
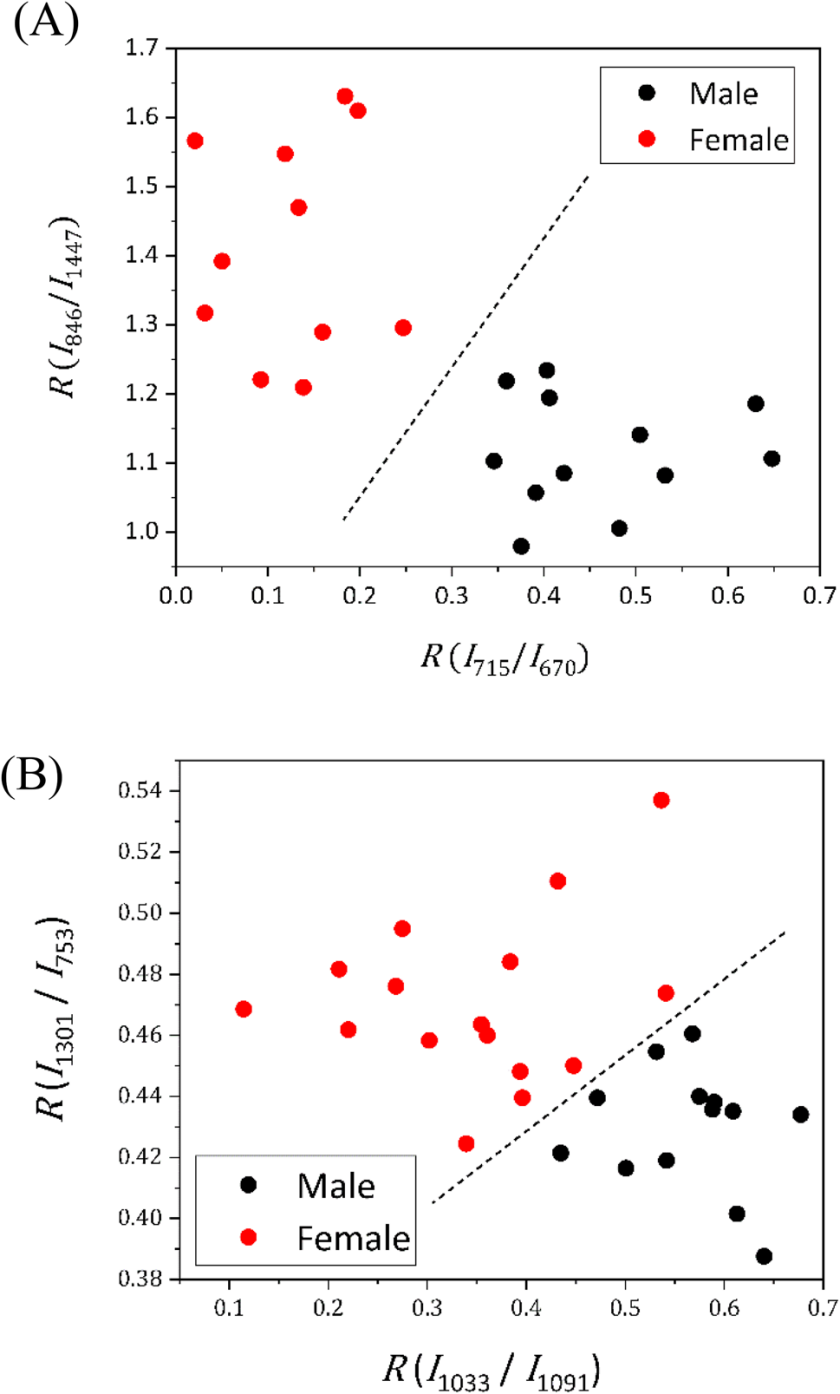
Plots of the ratio of spectral intensities defined as (**A**) (*x*, *y*) = (*R* (*I*_715_/*I*_670_), *R* (*I*_846_/*I*_1447_)) and (**B**) (*R* (*I*_1033_/*I*_1091_), *R* (*I*_1301_/*I*_753_)).

Furthermore, four Raman bands were identified for chick sexing based on their minimal *p*-value via *t*-test at 1447, 846, 715, and 670 cm^−1^. The corresponding *p*-values are shown in Table 4A. Box plots illustrating the band intensities according to chick sex are shown in Fig. S5. Intensities at 846 and 670 cm^−1^ were higher for females compared to males, while those at 1447 and 715 cm^−1^ exhibited stronger intensity for males compared to females. To elucidate the spectral differences depending on chick sex, intensities were plotted in two dimensions as (*x*, *y*) = (*I*_715_, *I*_670_) and (*I*_1447_, *I*_846_), combining two wavenumbers with higher intensities for males and females, respectively (Fig. S6). These plots were roughly categorized into male and female groups for (*I*_1447_, *I*_846_), and explicitly for (*I*_715_, *I*_670_). Furthermore, to emphasize the differences between males and females, intensity ratios were calculated as [*R* (*I*_715_/*I*_670_), *R* (*I*_846_/*I*_1447_)], where the spectral intensity stronger for males was divided by that weaker for males, and vice versa (Fig. 8A). The delineation to separate the plots into two groups based on sex can be easily drawn, as shown in Fig. 8A, enabling 100% accuracy in chick sexing.

**Table 4:** List of *p*-values for selected wavenumber for (A) RBCs and (B) whole blood.

Wavenumber (cm^−1^)	1447	1091	1033	756
(A)				
*p*-value	3.8 × 10^–5^	8.3 × 10^–5^	7.0 × 10^–8^	2.2 × 10^–6^

Wavenumber (cm^−1^)	1301	1091	1033	756
(B)				
*p*-value	1.7 × 10^–3^	2.8 × 10^–4^	3.3 × 10^–5^	1.8 × 10^–4^

Similarly, this method for discriminating chick sex using key wavenumbers with significant differences between sexes was applied to the dataset of Raman spectra obtained from whole blood. Four wavenumbers with the smallest *p*-value were selected: 1301, 1091, 1033, and 756 cm^−1^. The *p*-values are summarized in Table 4B. Box plots of the spectral intensity at these wavenumbers are shown in Fig. S7. The former two intensities were higher for females compared to males, while the latter two intensities showed the opposite trend. Plots of the intensities defined as (*x*, *y*) = (*I*_1033_, *I*_1091_) and (*I*_756_, *I*_1301_) are shown in Fig. S8. These plots showed a slight separation of the data based on chick sex. Although the plot of intensity ratios shown in Fig. 8B exhibited more distinct separation compared to mere spectral intensity, it was not as distinct as in the case of RBCs (Fig. 8A). This may be attributed to the presence of various foreign matters in whole blood aside from RBCs.

## Conclusion

This study showed the feasibility of chick sexing through blood analysis using Raman spectroscopy. Detailed analysis of Raman spectra from whole blood, RBCs, and blood plasma ascertained that chick sex could be discriminated based on the different ratios of oxy- and deoxy-RBC states using 785-nm excitation. Specifically, male Hb exhibited higher oxygen affinity, and spectral components attributed to oxy-RBCs, which were more prominent in males than females, were successfully extracted. By calculating the ratios of selected Raman bands characteristic for oxy- and deoxy-RBCs, chick sex could be discriminated with high accuracy, up to 100%. These results demonstrate the potential application of hematological traits in developing an automatic in ovo embryo sexing method through spectroscopic analysis.

### Supplementary Information

Supplementary Information.

## Data Availability

The data that support the findings of this study are available from the corresponding author upon reasonable request.
