# Supplementary material for: Chick sexing based on the blood analysis using Raman spectroscopy
Source: Sci Rep. 2024 Jul 11;14:15999. doi: 10.1038/s41598-024-65998-y (PMC11237000; doi:10.1038/s41598-024-65998-y)
Supplement: Supplementary file 1 — Supplementary Information. [file 41598_2024_65998_MOESM1_ESM.pdf]

Supplementary Information

Chick sexing based on the blood analysis using Raman spectroscopy

Sana Matsumoto[1], Akane Ogino[2], Kai Onoe[2], Juichiro Ukon[3], Mika Ishigaki[1]*

[1]*Institute of Agricultural and Life Sciences, Academic Assembly, Shimane University, 1060 Nishikawatsu, Matsue, Shimane, 690-8504, Japan*

[2]*NABEL Co., Ltd. 86 Morimoto-cho, Nishikujo, Minami-ku, Kyoto, 601-8444, Japan*

[3]*UKON Craft Science Ltd. 106-4, Fukakusa-Shhinmon-Jotyo, Fushimi-ku, Kyoto, 612-8436, Japan*

*Authors to whom correspondence should be sent.

*E-mail: ishigaki@life.shimane-u.ac.jp (M.I.)

Table of Contents

SI 1. Selection of the excitation wavelength for chick sexing and variations in Raman spectra of oxy- and deoxy-RBCs based on chick sex

SI 2. Figures

SI 1. Selection of the excitation wavelength for chick sexing

The selection of the excitation wavelength appropriate for chick sexing was performed first. Heparinized blood samples collected from the chick hearts (six males and six females) were measured using three excitation wavelengths: 532, 633, and 785 nm. To prevent the thermal damage by laser irradiation, Raman measurements were carried out with one to several drops of physiological saline. Each sample yielded two Raman spectra, and the mean Raman spectra were calculated after baseline correction and normalization based on the spectral area in the 1800–600 cm^{-1} region. Details of the measurement conditions are summarized in Table S1.

Overwritten Raman spectra of chick blood samples obtained using 532-nm, 633-nm, and 785-nm excitation wavelengths are shown in Figures S1A, S1B, and S1C, respectively. PCA analysis was conducted for the dataset obtained using each excitation wavelength. The dataset for a 532-nm excitation did not exhibit separation based on chick sex. For the 633-nm excitation, although the data tended to group slightly into two clusters, they overlapped each other (Figure S2). Conversely, with the 785-nm excitation, the data were distinctly split into two groups. Thus, the 785-nm excitation wavelength was selected for this study. Because the discussion on chick sexing using the 785-nm excitation is provided in the main text, further details are omitted here.

To investigate why the 785-nm excitation wavelength is more appropriate than the other two excitation wavelengths for chick sexing, the resonance spectra of RBCs were studied. The 532-nm excitation wavelength matches the electronic absorption band of oxy- and deoxy-Hb [13-18], facilitating the selective recording of resonance Raman spectra of Hb. Thus, the most representative resonance Raman spectra of oxy-RBCs and deoxy-RBCs were recorder using a 532-nm excitation wavelength.

Figure S3 shows the Raman spectra of oxy- and deoxy-RBCs measured using (A) 532-nm and (B) 785-nm excitations. At 532 nm, the spectra of oxy- and deoxy-RBCs showed markedly different spectral patterns depending on the distinct states of RBCs. To get RBCs oxygenated or deoxygenated in this study, oxygen or nitrogen gas was introduced into the RBC samples for several minutes. Thus, the spectral variations in oxy- and deoxy-RBCs may be attributed to the degree of oxygen saturation. The

reduced spectral variations in deoxy-RBCs compared to oxy-RBCs can be attributed to the ease with which bonds between Hb and oxygen atoms are broken under low oxygen concentrations, leading to complete deoxygenation of RBCs. The spectral variations depending on the different states of RBCs measured using a 785-nm excitation can also be discussed in terms of oxygen saturation (Figure S3B). However, at 785 nm excitation, the spectral variations of oxy-RBCs were not prominent. The intensities in the Raman spectra of oxy-RBCs appeared to be lower than those of deoxy-RBCs because the absorbance of the near-infrared light at approximately 785 nm by oxy-Hb is lower than those by deoxy-Hb [15, 16].

The appropriateness of the 785-nm excitation wavelength for chick sexing is now summarized. The UV–vis absorbances at ~532 nm attributed to two types of Hb were comparable [14, 18]. Thus, strong Raman spectra were obtained from both oxy- and deoxy-RBCs via resonance effect with Hb. The considerable spectral variations, especially in the 1650–1600 and 1400–1340 cm^{-1} regions, among spectra obtained using a 532-nm excitation appeared to sensitively reflect the different ratios of oxy- and deoxy-RBC states (Figure S1A). Spectral intensities were normalized based on the area in the 1800–600 cm^{-1} region. Thus, substantial spectral variations attributed to deoxy-RBCs notably affected the spectral intensities, potentially overshadowing variations related to chick sex.

At 633 nm, strong Raman signals from RBCs were obtained via preresonance effect. Especially, the absorbance of the visible light attributed to deoxy-Hb notably surpasses that of oxy-Hb, leading to strong detection of the Raman signal from deoxy-RBCs [14, 18]. This wavelength is commonly used in oximeters for effectively detecting visible light absorption caused by deoxy-Hb [15, 16]. Spectral variations associated with the 633 nm excitation wavelength were smaller compared to a 532-nm excitation (Figures S1A and S1B). This suggests that the Raman spectra for blood samples largely reflect deoxy-RBCs, with the contribution of blood spectral components owing to oxy-RBCs almost obscured, making differences between males and females almost undetectable.

At ~785 nm, the resonance effect does not sufficiently operate at this

wavelength [14, 18]. Thus, the other blood components such as proteins and phospholipid comparably contribute to the spectra of RBCs, and the small spectral variations owing to the contribution of oxy- and deoxy-Hb are detected in the 1650–1500 and 1300–1200 cm^{-1} regions. Therefore, this wavelength is considered suitable for discriminating chick sex because the ratio between oxy- and deoxy-RBCs appears to differ between males and females, and these different ratios are detectable. Namely, the only signals due to RBCs enhanced by resonance and preresonance effect are expected to be insufficient to determine the sex of the chicks, and too strong spectral variations caused by oxygenation and deoxygenation of RBCs also disturb to determine the sex.

Table S1: Summary of measurement conditions for chick blood samples using three excitation wavelengths.

**Wavelength (nm)**	**532**	**633**	**785**
**Grating (lines/mm)**	1800	600	600
**Raman shift (cm^{-1})**	1900 - 400	1900 - 400	1900 - 400
**Measurement time**	30 s × 10	20 s × 4	30 s × 10
**Number of measurement times**	2	3	2
**Laser power (mW)**	3	3	40
**Objective lens**	60	100	60

(A)

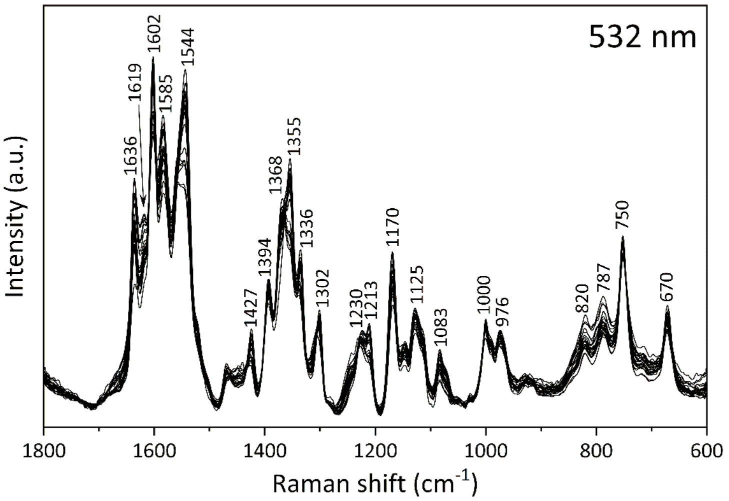

532 nm
1636
1619
1602
1585
1544
1368
1355
1336
1394
1427
1302
1230
1213
1170
1125
1083
1000
976
820
787
750
670
Intensity (a.u.)
Raman shift (cm-1)
1800
1600
1400
1200
1000
800
600

(B)

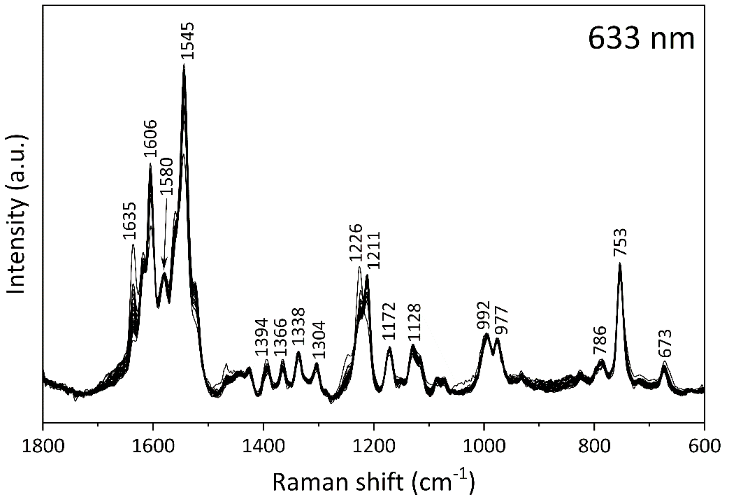

633 nm
1635
1606
1580
1545
1394
1366
1338
1304
1226
1211
1172
1128
992
977
786
753
673
Intensity (a.u.)
Raman shift (cm-1)
1800
1600
1400
1200
1000
800
600

(C)

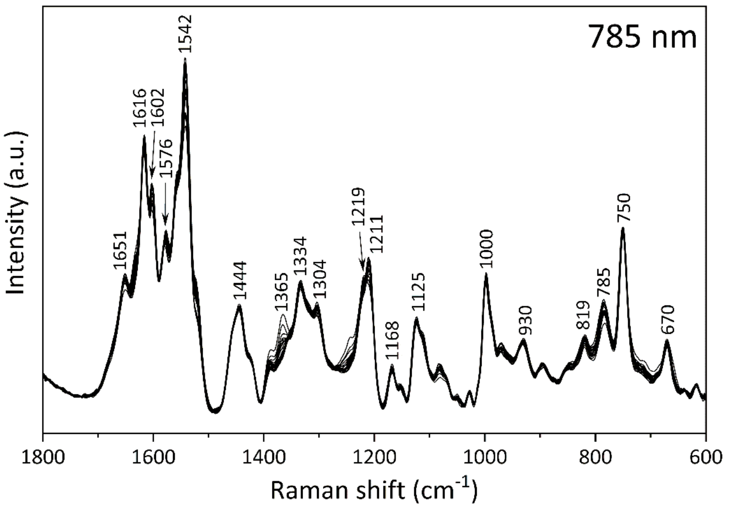

785 nm
1651
1616
1602
1576
1542
1444
1365
1334
1304
1219
1211
1168
1125
1000
930
819
785
750
670
Intensity (a.u.)
Raman shift (cm-1)
1800
1600
1400
1200
1000
800
600

Figure S1: Overwritten Raman spectra of whole blood obtained from six males and six females using (A) 532-nm, (B) 633-nm, and (C) 785-nm excitation wavelengths.

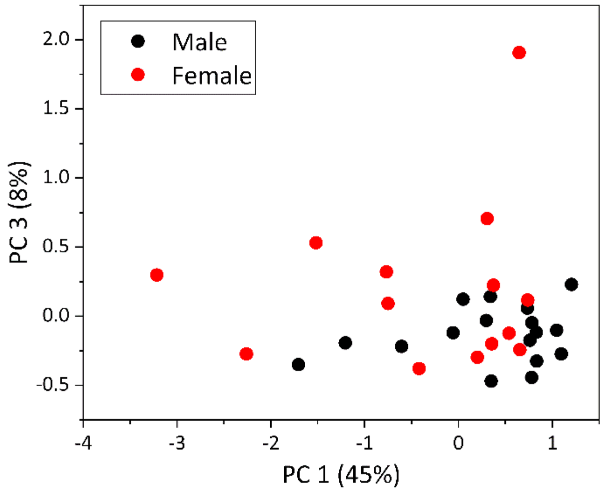

Figure S2: Score plots of PC 1 vs. PC 3 from PCA performed on the Raman spectral dataset obtained using a 633-nm excitation.

(A)

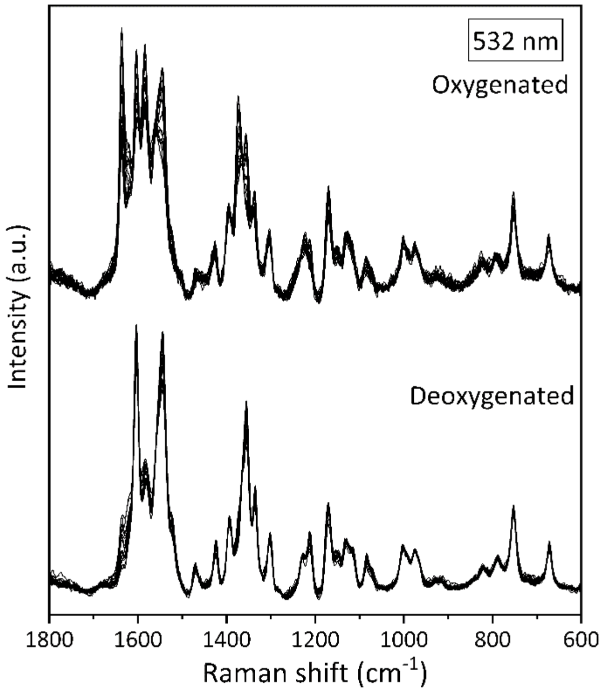

(B)

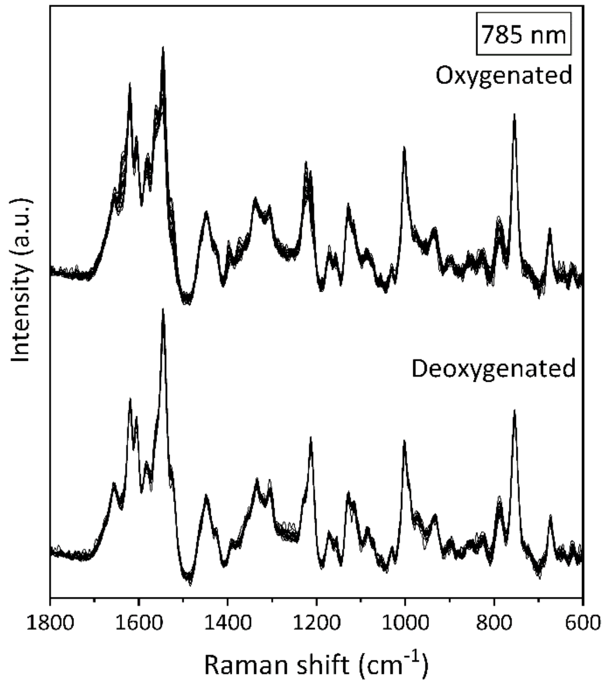

Figure S3: Raman spectra of oxy- and deoxy-RBCs using (A) 532-nm and (B) 785-nm excitation wavelengths.

SI 2. Figures

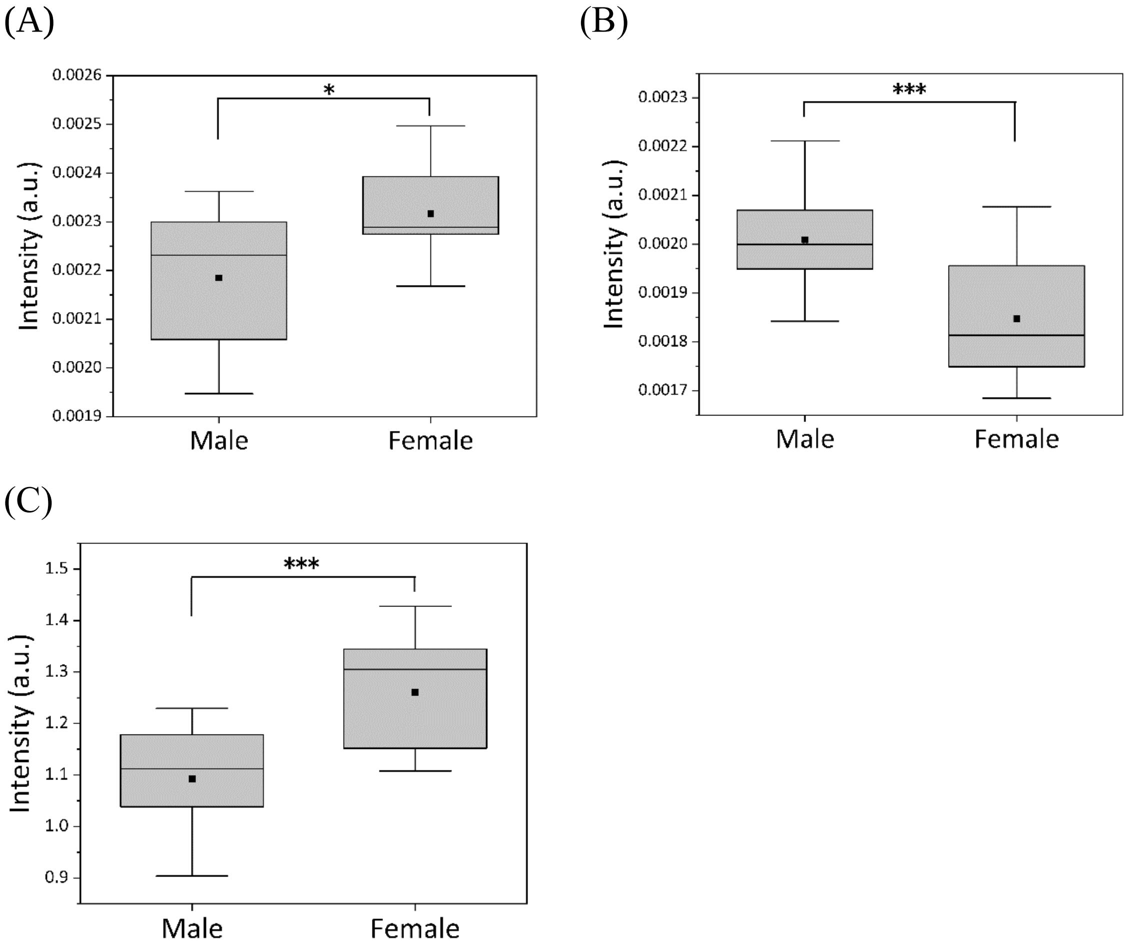

Figure S4: Box plots of band intensities at (A) 787 cm^{-1} and (B) 1640 cm^{-1}, along with the ratio of band intensity defined as (C) I_{787}/I_{1640}.

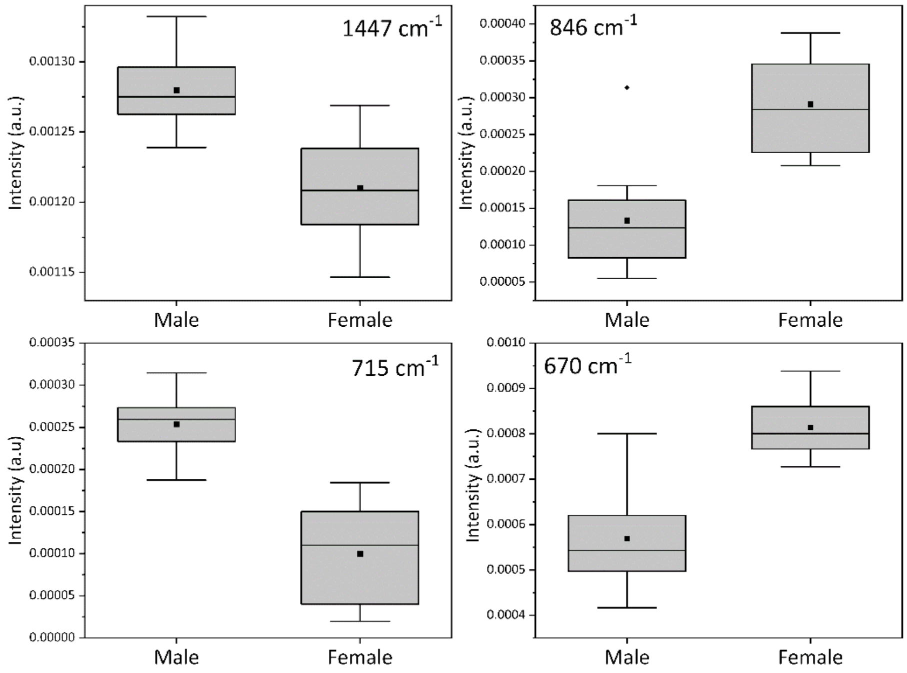

Figure S5: Box plots of the four spectral intensities for RBCs with the least *p*-values from *t*-tests.

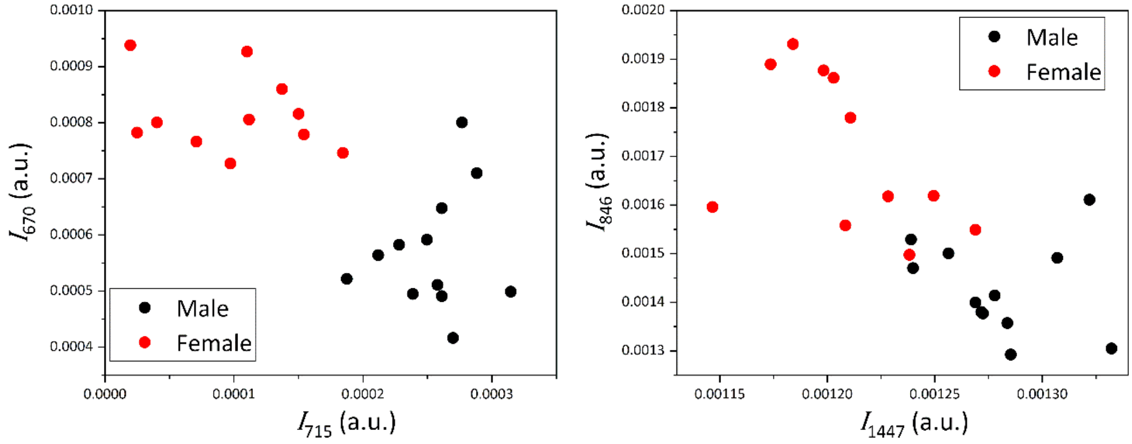

Figure S6: Plots of spectral intensities as $(x, y) = (I_{715}, I_{670})$ and (I_{1447}, I_{846})

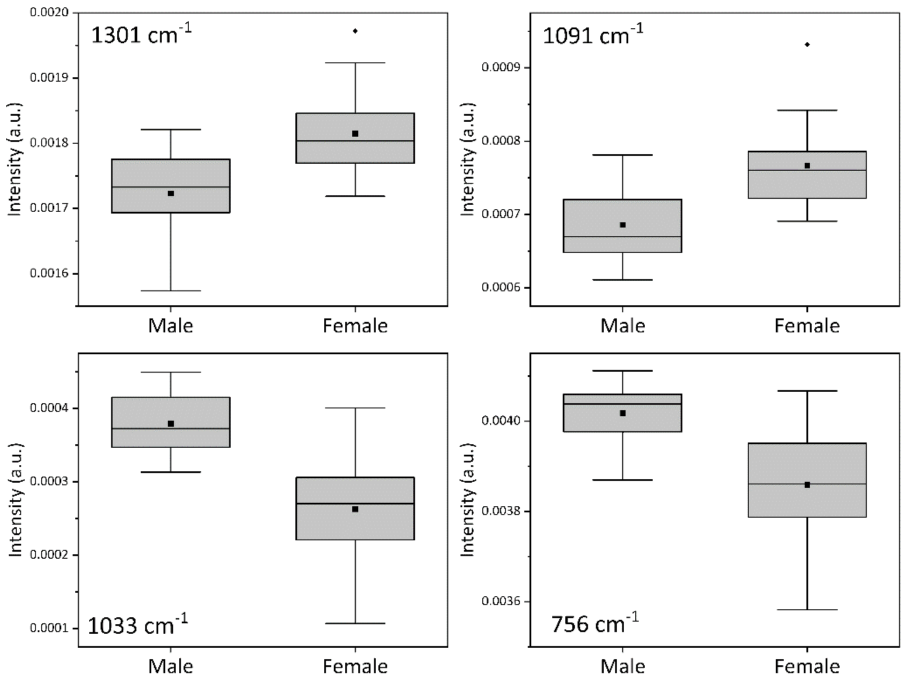

Figure S7: Box plots of the four spectral intensities for whole blood samples with the least p-values from t-tests.

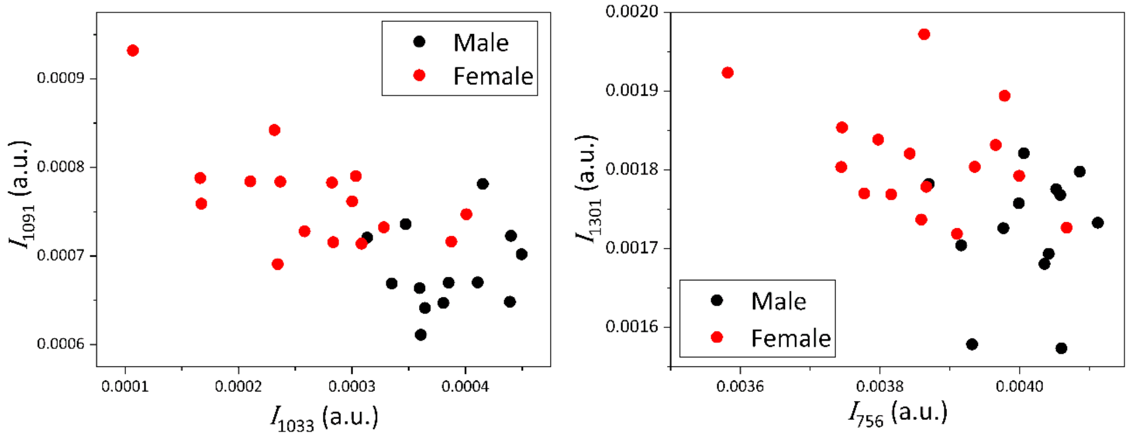

Figure S8: Plots of spectral intensities defined as $(x, y) = (I_{1033}, I_{1091})$ and (I_{756}, I_{1301}).
